# Comparison of the Effect of Combination of Triamcinolone Acetonide and Vitamin A Mouthwash with Triamcinolone Mouthwash Alone on Oral Lichen Planus

**DOI:** 10.5681/joddd.2010.006

**Published:** 2010-03-14

**Authors:** Zohreh Dalirsani, Ali Taghavi Zenouz, Masoumeh Mehdipour, Fakhri Alavi, Yousef Javadzadeh

**Affiliations:** ^1^ Assistant Professor, Department of Oral Medicine, Faculty of Dentistry, Mashhad University of Medical Sciences, Mashhad, Iran; ^2^ Assistant Professor, Department of Oral Medicine, Faculty of Dentistry, Tabriz University of Medical Sciences, Tabriz, Iran; ^3^ Post-graduate Student, Department of Oral & Maxillofacial Radiology, Faculty of Dentistry, Tabriz University of Medical Sciences, Tabriz, Iran; ^d^ Associate Professor, Department of Medicinal Chemistry, Faculty of Pharmacy, Tabriz University of Medical Sciences, Tabriz, Iran

**Keywords:** Mouthwash, oral lichen planus, triamcinolone, vitamin A

## Abstract

**Background and aims:**

Lichen planus is a relatively common mucocutaneous disease, with an unknown etiology. There is no complete cure for oral lichen planus (OLP), but some drugs, including corticosteroids, retinoids, cyclosporine and antibiotics are commonly used for treatment of OLP. The aim of the present study was to compare the effect of combi-nation of triamcinolone and vitamin A mouthwash with triamcinolone mouthwash alone on OLP.

**Materials and methods:**

Twenty OLP patients were randomly divided into two groups of 10. The experimental group was treated with combination of triamcinolone and vitamin A mouthwash and the control group was treated with triamcinolone-containing mouthwash alone. The patients were examined once every two weeks and the lesion size, pain and burning sensation were recorded based on visual analogue scale during a two-month period. Data were analyzed by Mann-Whitney U test using SPSS software.

**Results:**

The use of combination of triamcinolone-vitamin A mouthwash was effective in decreasing the pain and burning sensation of OLP (P = 0.012). Decrease in pain and burning sensation were similar in both groups (P = 0.73). The use of combination of triamcinolone-vitamin A mouthwash led to a decrease in the size of keratotic, atrophic and erosive OLP lesions (P = 0). Decrease in the size of the lesions was significantly greater in the study group compared to the control group (P = 0.029).

**Conclusion:**

The use of combination of triamcinolone-vitamin A mouthwash is effective in decreasing the size of keratotic, atrophic or erosive lesions.

## Introduction

Oral lichen planus (OLP) is a chronic inflammatory disease with an 0.5-2.2% prevalence rate in the general population.^1^ Oral lesions in OLP are chronic and rarely undergo spontaneous remission. The cause of OLP is unknown, although several factors have been considered, including: stress, diabetes, hepatitis C, hypersensitivity to metals, genetic predisposition and anxiety.^2^ Currently there is no known cure for this disease, but some drugs such as topical and systemic corticosteroids, topical and systemic retinoids, cyclosporine and antibiotics are commonly used for treatment of OLP.^1^

The use of systemic corticosteroids is limited by their side effects.^3^ The greatest problem of using topical corticosteroids in the oral cavity is to make them adhere to the mucosa for a sufficient period of time. That is why potent topical corticosteroids in adhesive pastes are the safest and the most effective treatment for OLP^4^ for long-term use without systemic side effects.

Systemic and topical retinoids have been used to treat OLP.^5^ Retinoids have anti-inflammatory properties, perhaps through their interactions with the arachidonic acid cascade. Retionoids have been noted to have anti-keratinizing and immuno-modulating effects; they stimulate macrophage activation and antibody-dependent cell-mediated cytotoxicity.^6^ Moreover, retinoids may reduce CD4 lymphocyte infiltration and increase macrophages in OLP lesions, thus accelerating the healing process.^7^ Therefore, retinol and its synthetic and natural analogues (retinoids) may be useful in the treatment of OLP.^6^ Unfortunately, the systemic use of retinoids is often limited due to their side effects, too.^6^ Therefore, topical retinoids have been developed and found to produce generally good results in patients with OLP.^6^ Topical retinoid has a reported immuno-modulating effect on OLP,^7^ and has been successfully used to treat OLP in some cases where corticosteroids have failed to achieve satisfactory results.^8^

Topical retinoids and corticosteroids have shown promising effects in the treatment of OLP.^7 , 9^ Retinoic acid rinse has not yet been compared with triamcinolone acetonide mouthwash in clinical trials in Iranian patients. The purpose of this study was to compare the efficacy of retinoic acid rinse with triamcinolone acetonide mouthwash for the treatment of erosive and atrophic OLP.

## Materials and methods

A randomized clinical trial was designed to evaluate the effect of combination of triamcinolone-vitamin A mouthwash on OLP. In this double-blind study, patients with erosive and atrophic OLP referring to the Department of Oral Medicine at Faculty of Dentistry, Tabriz University of Medical Sciences were asked to participate in the study. OLP had been confirmed through clinical examination, biopsy and histopathological evaluation.

All the patients were interviewed for a thorough medical history. We explained the study procedure to the patients; then they filled out informed written consent forms. The subjects having the inclusion criteria were chosen for the study.

### Inclusion criteria

1. Patients with oral lichen planus (confirmed by clinical examination, biopsy and histopathological evaluation) with or without skin involvement
2. Patients with keratotic and erosive and/or atrophic lesions

### Exclusion criteria

1. Contraindications of corticosteroid use; patients under 18 years of age; uncontrolled diabetes; pregnant or breast-feeding women; immunodeficiency or HCV infection
2. Patients with lichenoid reaction potential as evidenced by histopathological examination; drug intake or other predisposing conditions
3. Patients exhibiting dysplasia in histopathological evaluation
4. Patients who had received any medications for lichen planus during the past month
5. Known allergy to retinoids and triamcinolone

The drugs for this study were 0.2% triamcinolone acetonide mouthwash and combination of triamcinolone acetonide and vitamin A mouthwash, which were prepared in the Department of Medicinal Chemistry in the Faculty of Pharmacy at Tabriz University of Medical Sciences. In this double-blind study, 20 patients were divided into 2 groups (experimental and control) according to Block Randomization (www.randomization.com).

In the experimental group, the subjects (N = 10) were treated with combination of triamcinolone acetonide and vitamin A mouthwash (group 1) three times daily for one month. In the control groups, the subjects (N = 10) received 0.2% triamcinolone acetonide mouthwash (group 2).

The patients were evaluated for possible side effects of the medication, one week after the initiation of the study and afterwards were regularly examined every 2 weeks for 2 months. Location, size of the lesions, and pain sensation in each patient were recorded in the questionnaires. A 10-point visual analogue scale (VAS)^1^ was used for assessing pain severity induced by the lesion.^10^ Digital callipers were used for evaluating lesions size. Visual analogue scale score and lesion size were recorded every session. Data were analyzed by Mann-Whitney U test using SPSS 13.0 computer software.

## Results

Twenty patients with keratotic and atrophic/erosive OLP participated in the study. The mean age of the patients was 40 ± 2 years old.

**Figure 1:**
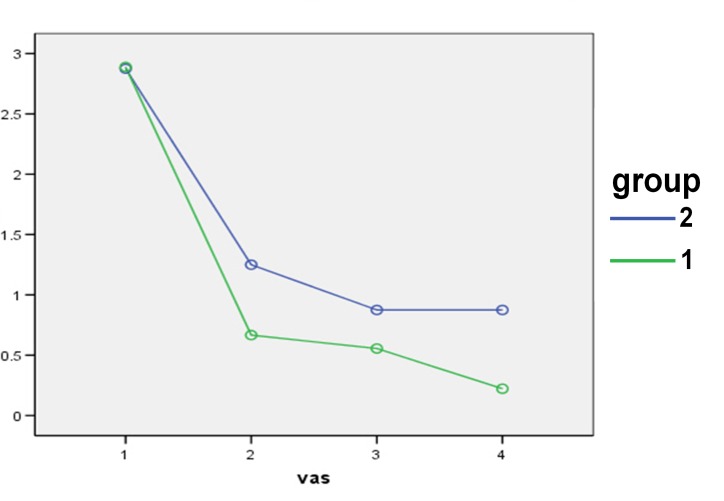
Comparison of decrease in pain severity between the two groups.

The use of medications in the two groups led to a decrease in pain and burning sensation severity of OLP, which was statistically significant (P = 0.012 and P = 0.012, respectively). The difference between the two groups was not statistically significant (P = 0.73) (Figure 1).

**Figure 2:**
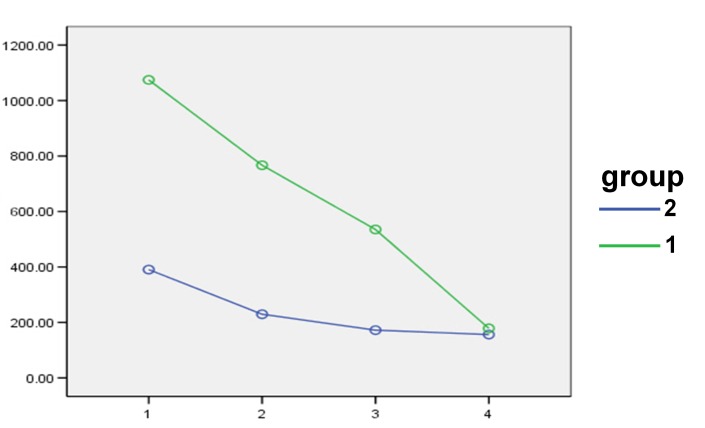
Comparison of decrease in the size of keratotic lesions between the two groups.

The use of combination of triamcinolone acetonide and vitamin A mouthwash and triamcinolone mouthwash alone led to a decrease in the size of keratotic lesions, which was statistically significant (P = 0.001 and P = 0.001, respectively). The decrease in the size of keratotic lesions was greater in the experimental group compared to the control group. (P = 0.03) (Figure 2).

**Figure 3:**
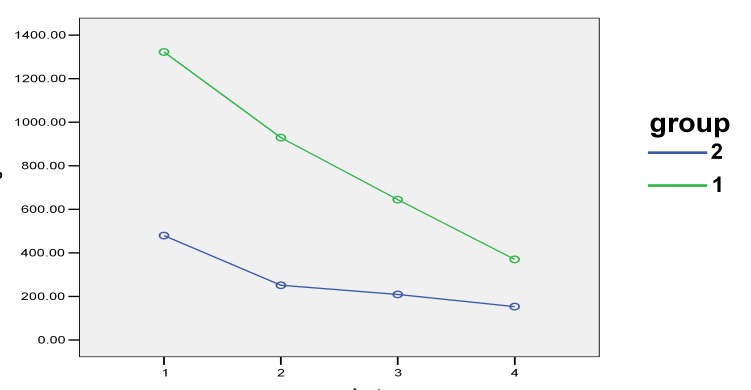
Comparison of decrease in the size of erosive/atrophic lesions between the two groups.

The use of medications in the two groups led to a decrease in the size of keratotic and atrophic/erosive lesions OLP lesions, which was statistically significant in both groups (P = 0.0 and P = 0.0, respectively). The decrease in the size of keratotic and atrophic/erosive lesions was greater in the experimental group compared to the control group (P = 0.029) (Figure 3).

## Discussion

This study revealed that combination of 0.2% triamcinolone acetonide and vitamin A mouthwash and triamcinolone acetonide mouthwash alone were effective in reducing lesion size, pain and irritation of oral erosive lichen planus.

According to the results of the present study, there were no significant differences in pain severity and burning sensation decrease between the experimental and control groups.

The results of the present study are consistent with a previous study, evaluating the efficacy of 0.2% triamcinolone acetonide mouthwash in the treatment of OLP, which reported that topical triamcinolone acetonide reduces the severity of the pain and burning sensation of atrophic and erosive OLP lesions.^11^

In our study application of triamcinolone acetonide mouthwash decreased pain severity in almost 75% of patients while in that study the decrease in pain severity was observed in 86.7% of patients. Furthermore, our results supported the idea that triamcinolone acetonide-vitamin A mouthwash is more effective than triamcinolone acetonide mouthwash alone on the keratotic lesions.

An investigation on the effect of 0.5% triamcinolone acetonide mouthwash on erosive OLP lesions revealed that a total of 57% of patients demonstrated complete response in ulceration size after 3 months;^12^ however, in the present study, there was a 25% decrease in the size of ulcerated lesions in the control group after treatment. This difference in results might be attributed to the use of 0.2% triamcinolone acetonide mouthwash instead of 0.5% triamcinolone acetonide mouthwash in the present study and differences in the duration of treatment.

A study on intra-lesional triamcinolone acetonide injection and its efficacy in treating ulcerative OLP reported that 84.4% of patients demonstrated complete response in ulceration size.^13^ The latter study concluded that intra-lesional injection of triamcinolone acetonide in ulcerative OLP was effective and safe in achieving lesion and pain regression. The difference of that study with the present study, which showed a 25% improvement rate, might be attributed to different formulations and different treatment methods.

Evaluating the efficacy of isotretinoin on OLP for 4 months, Piattelli et al^5^ reported 50% complete and 50% partial responses. In the present study, the complete improvement of lesion size was 10%. This difference might be attributed to differences in treatment duration. Another studyl showed that OLP lesions improved in 94% of patients following the application of isotretinoin gel therapy,^14^ which is consistent with our results with 100% of patients showing partial or complete recovery of lesions. More improvements in lesions might be attributed to combination of triamcinolone acetonide with vitamin A. However, more clinical trials with greater sample sizes are recommended.

## Conclusion

Combination of triamcinolone acetonide and vitamin A mouthwash might be significantly more effective than triamcinolone acetonide mouthwash alone in decreasing the size of OLP lesions. This treatment method could be used in OLP treatment.
